# Influence of Low Molecular Weight Salts on the Viscosity of Aqueous-Buffer Bovine Serum Albumin Solutions

**DOI:** 10.3390/molecules27030999

**Published:** 2022-02-01

**Authors:** Blaž Zdovc, Matej Jaklin, Barbara Hribar-Lee, Miha Lukšič

**Affiliations:** Faculty of Chemistry and Chemical Technology, University of Ljubljana, Večna Pot 113, SI-1000 Ljubljana, Slovenia; blaz.zdovc@ki.si (B.Z.); matej.jaklin@fkkt.uni-lj.si (M.J.); barbara.hribar@fkkt.uni-lj.si (B.H.-L.)

**Keywords:** bovine serum albumin, viscosity, zeta-potential, ion-specific effects, protein aggregation

## Abstract

Pharmaceutical design of protein formulations aims at maximum efficiency (protein concentration) and minimum viscosity. Therefore, it is important to know the nature of protein-protein interactions and their influence on viscosity. In this work, we investigated the dependence of the viscosity of BSA in an aqueous 20 mM acetate buffer at pH = 4.3 on protein concentration and on temperature (5–45 °C). The viscosity of the solution increased with protein concentration and was 230% higher than the viscosity of the protein-free formulation at 160 mg/mL. The viscosity decreased by almost 60% in the temperature range from 5 to 45 °C. The agreement of the modified Arrhenius theory with experiment was quantitative, whereas a hard-sphere model provided only a qualitative description of the experimental results. We also investigated the viscosity of a 100 mg/mL BSA solution as a function of the concentration of added low molecular weight salts (LiCl, NaCl, KCl, RbCl, CsCl, NaBr, NaI) in the range of salt concentrations up to 1.75 mol/L. In addition, the particle size and zeta potential of BSA-salt mixtures were determined for solutions containing 0.5 mol/L salt. The trends with respect to the different anions followed a direct Hofmeister series (Cl^−^ > Br^−^ > I^−^), whereas for cations in the case of viscosity the indirect Hofmeister series was observed (Li^+^ > Na^+^ > K^+^ > Rb^+^ > Cs^+^), but the values of particle sizes and zeta potential did not show cation-specific effects. Since the protein is positively charged at pH = 4.3, anions are more attracted to the protein surface and shield its charge, while the interaction with cations is less pronounced. We hypothesize that salt surface charge shielding reduces protein colloidal stability and promotes protein aggregate formation.

## 1. Introduction

Bovine serum albumin (BSA) is a protein found in bovine blood serum. One of its main features is specific binding sites that have physiological significance for transport, modulation and inactivation of metabolite and drug activities [1,2,3,4]. BSA is a universal carrier for fatty acids, ions, ligands, hormones and metabolites [5,6]. It is a large heart-shaped (elliptical) globular protein consisting of 607 amino acids and having a molecular weight of 66.43 kDa [7]. Its isoionic point is estimated to be around 4.7, which means that the protein molecule has a net negative charge under physiological conditions (pH = 7.4) [8,9] (the value of the isoionic point depends on both the composition of the solution and the method of determination; values between 4.6 and 5.1 have been reported in the literature [10]). The secondary structure of the protein is reversible up to 45 °C and beyond this temperature BSA is thermally denatured [11]. Because of its relatively inexpensive production and its similarity to human serum albumin (HSA), BSA is widely studied as a model globular protein [5].

The viscosity of protein solutions is the result of various interactions between protein molecules in the solution. These interactions are divided into two groups: attractive and repulsive. In the category of repulsive interactions, we include the long-range Coulomb repulsive interactions between like charges and the excluded volume effect, which is a short-range interaction. The attractive interactions in protein solutions are the Coulomb interactions between opposite charges, van der Waals interactions, hydrogen bonding, and hydrophobic contact. In protein formulations, protein-protein interactions depend mainly on long-range repulsive Coulomb interactions. If we take into account all the effects that can occur in protein solutions, we can see that the viscosity of protein solutions is a complex function of the individual contributions of the various interactions [12]. For solutions with rather low concentrations of BSA (<40 mg/mL [13,14]), the viscosity is determined primarily by the electroviscous effects [15]. Among these, three contributions can be accounted for: (1) the diffuse double layer around the protein molecule imposes resistance to the flow, (2) there is repulsion between the double layers and (3) the protein-protein interactions can affect the shape of the protein molecules. Therefore, viscosity is minimal at a pH corresponding to the isoionic point of the protein. At pH values where the protein carries a net charge (positive or negative), the repulsive electrostatic interactions between protein molecules lead to an increase in viscosity. At high concentration (>200 mg/mL in the case of BSA [14]), however, short-range attractive interactions begin to predominate. They become effective because in concentrated solutions the distance between particles has decreased significantly. The short-range interactions become most evident at pH = pI, leading to the formation of soluble aggregates and increasing the viscosity compared to conditions with pH < pI or pH > pI.

Interaction of the low molecular weight salts or buffer species with the protein surface may also contribute to the solution’s flow [16]. In many aqueous-buffer protein solutions, the interaction of various salt ions with the protein molecule is salt specific [17,18,19,20,21,22,23]. Because of their high polarizability, kosmotropic ions bind the hydration water more tightly, causing protein molecules to prefer the hydrophobic protein-protein interactions in solution. This effect is called salting-out and decreases the solubility of the protein. Chaotropic ions have low polarizabilities, and preferentially bind in the layer between water and protein molecules, resulting in destabilization of the protein molecules. This effect is called salting-in and increases the solubility of the protein in solution [24,25,26]. Various ions have been assembled into a so-called Hofmeister series. In most cases, the inverse Hofmeister series applies for anions (F^−^ < Cl^−^ < Br^−^ < I^−^) and a direct Hofmeister series is characteristic for cations (Li^+^ < Na^+^ < K^+^ < Rb^+^ < Cs^+^) [8]. Previous studies have shown that the viscosity of the aqueous-buffer BSA solution increases with increasing concentration of protein at constant temperature [11]. In the same study, the viscosity of the BSA solution was also shown to decrease with increasing temperature at constant concentration of protein in the solution [11,27]. In examining the salt-specific effects on BSA, previous studies have shown that the diffusion coefficient for anions follows an inverse Hofmeister series, whereas the trend for cations does not follow a monotonic series, but rather a “bell-shaped” trend, where we can see the highest value for the diffusion coefficient of the protein in the BSA solution with added KCl and RbCl salts, then NaCl, LiCl, and the lowest value for the diffusion coefficient of the protein in the BSA solution with added CsCl salt [8]. In this paper, we first address the excluded volume effect on the viscosity of BSA solutions. We have studied the dependence of the viscosity, η, of BSA in aqueous 20 mM acetate buffer (pH = 4.3) at 25 °C on protein concentration up to 160 mg/mL. The effect of temperature on steric repulsion between protein molecules was considered, and for a protein concentration of 100 mg/mL, the dependence of η on temperature was measured in the range from 5 to 45 °C. We modelled the concentration and temperature dependence of the viscosity of the solution within the framework of absolute rate theory using a modified Arrhenius equation. This model has already been shown to agree well with experiments [11]. We have also modelled the dependence of the viscosity of the solution with a hard-sphere model and compared it with the modified Arrhenius-type model to evaluate the excluded volume effect on the viscosity. Next, we investigate how the viscosity of the BSA solution can be controlled by the addition of a low molecular weight salts. This paper sheds light on how the chemical nature of salt ions affects protein-protein interactions in highly concentrated protein formulations. The added electrolyte affects the electrostatic interactions between protein molecules, which can lead to protein-protein repulsion or attraction. Due to the high protein and ion concentration, the colloid stability of the solution may decrease and aggregates may form [22,23]. Since aggregates affect the viscosity of the solution, we quantified the colloid stability by also measuring their zeta potentials and particle sizes. The different degree of colloid stability of different simple salts could also give us information about their specific interaction with protein molecules. We systematically address the ion-specific trends of halide anions and alkali metal cations. The low molecular weight salts selected in this work are alkali metal cations and halide anions that are present in the human body, so that the human immune system does not react to them as intruders when the protein formulations are injected into the body. In addition to electrostatic interactions, chaotropic ions (e.g., I^−^, Cs^+^) can also mediate the so-called hydrophobic interactions that cause a decrease in the viscosity of the solution. (i) We have shown the dependence of the viscosity of 100 mg/mL BSA solutions on the concentration of the added low molecular weight salts (LiCl, NaCl, KCl, RbCl, CsCl, NaBr, NaI) in the range of salt concentrations up to 1.75 mol/L at 25 °C and (ii) on the temperature in the range of 5 to 45 °C at a salt concentration of 1 M. The charges of the low molecular weight salt ions are rather small, but can still affect the net charge of the protein molecules. The influence of the addition of electrolyte co-solutes (which are also responsible for the osmotic balance of the formulation) on the viscosity of protein solutions is of great practical importance in pharmaceutical and separation sciences. In addition, our work provides experimental data for the further development and application of statistical mechanical theories used to predict the viscosity of globular protein solutions.

The paper is organized as follows: After the introduction mentioned above, in the theoretical part we describe the modified Arrhenius-type model and the hard-sphere model for the viscosity of protein solutions. In the experimental part, we describe the chemicals and equipment used and the preparation of the solutions. The results of our work are described and discussed in the Results and Discussion section, and the conclusions are summarized in the Conclusions section.

## 2. Theoretical Part

### 2.1. Hard-Sphere Model

Hard-sphere model is the most rudimentary model to describe particles in a solution. The solute is represented by a hard-sphere with a given radius, *r*, embedded in an implicit structure-less solvent. A. Einstein was the first to describe the relation between viscosity of a diluted solution, η, and volume fraction of the solute, ϕ, through Equation (1) [28]

(1) $$\eta \left(\phi \right)=\eta_{0}\left(1+\frac{5}{2}\phi \right)$$

where $\eta_{0}$ is the viscosity of the solvent (aqueous-buffer in our case) and $\phi =\left(4/3\right)\pi r^{3}cN_{A}/M_{p}$, where *c* is the concentration of the solute (protein in our case) in the solution, $N_{A}$ is the Avogadro constant and $M_{p}$ is the molecular weight of the solute (protein). To model the viscosity of more concentrated solutions, the expansion of Einstein’s Equation (1) for diluted solutions to the second order was proposed. For the description of BSA solutions we used the following expression (2) [28]:

(2) $$\eta \left(\phi \right)=\eta_{0}\left(1+\frac{5}{2}\phi +6.17\phi^{2}\right)$$

### 2.2. Absolute Rate Theory

A modified Arrhenius formula was used to model the concentration and temperature dependence of the viscosity. The relative viscosity, $\eta_{r}$, of the protein solution is given with Equation (3) [11]

(3) $$\eta_{r}=\frac{\eta }{\eta_{0}}=\exp\left[\frac{c}{\alpha -\beta c}\left(-\tilde{B}+T+\frac{\Delta \tilde{E}}{RT}\right)\right]$$

where η is the viscosity of protein solution, $\eta_{0}$ is the viscosity of the solvent (aqueous-buffer solution), *c* is the mass concentration of the protein, *T* is the absolute temperature and *R* is the gas constant. Parameter α is defined with Equation (4)

(4) $$\alpha =\rho_{b}\frac{M_{p}}{{\bar{M}}_{b}}$$

where $\rho_{b}$ is the density of aqueous-buffer solution, $M_{p}$ is the molecular weight of the protein and ${\bar{M}}_{b}$ is the average molecular weight of the solvent. It is worth noting that for dilute buffer solutions, ${\bar{M}}_{b}$ is practically identical to the molecular weight of water $M_{w}$. Parameter β is given with Equation (5)

(5) β=να−1

where ν is obtained from fitting the model function (3) to the experimental viscosity data. Viscosity of solvent is modelled with Equation (6)

(6) $$\eta_{0}=\exp\left[-B_{b}+D_{b}+\frac{\Delta E_{b}}{RT}\right]$$

By global non-linear least-squares regression of Equations (3) and (6) to experimental viscosity data, one obtains coefficients $B_{b}$, $D_{b}$, $B_{p}$, and $D_{p}$ coefficients and activation energies $\Delta E_{b}$ and $\Delta E_{p}$ for BSA solution (subscript “p”) and acetate buffer (subscript “b”). Parameters $\tilde{B}$, $\tilde{D}$, and $\tilde{E}$ in Equation (3) are defined with relations (7)

(7) $$\begin{array}{cc} \tilde{B} & =B_{p}-B_{b}\\ \tilde{D} & =D_{p}-D_{b}\\ \Delta \tilde{E} & =\Delta E_{p}-\Delta E_{b} \end{array}$$

## 3. Experimental Part

### 3.1. Chemicals and Solution Preparation

Bovine serum albumin (BSA, free of fatty acids, purity ≥96%) was purchased from Sigma Aldrich. Other chemicals (concentrated acetic acid, 1 mol/L aqueous NaOH, solid LiCl, NaCl, KCl, CsCl, and NaBr) were purchased from Merck, RbCl from Riedel-De Haën, and NaI from Sigma Aldrich. 20 mM aqueous acetate buffer solution was prepared with Mili Q water using acetic acid and NaOH. A given amount of acetic acid in water was titrated to pH 4.3 with 1 mol/L aqueous NaOH solution at room temperature. The pH was determined using an Iskra pH meter (model MA 5740) and a combined glass-microelectrode InLab 423 (Mettler Toledo). The buffer was used to prepare all other solutions (BSA, low molecular weight salt). All low molecular weight salts except LiCl were first dried at 105 °C for 3 h, while LiCl was dried at 130 °C for 3 h. The salts were left in the desiccator to cool. Stock solutions of the salts in acetate buffer were prepared gravimetrically and filtered through 0.45 μm Sartorius Ministart Syringe Filter. The BSA stock solution was prepared gravimetrically in acetate buffer. The solution was dialyzed extensively against the buffer solution using a dialysis membrane (Spectrum, Spectra/Por molecular porous membrane tube; Mw cut-off 3.5 kDa). The buffer was changed three times every 8 h. The concentration of the BSA stock solution was determined using the UV-VIS spectrophotometer (Agilent Technologies, Cary 100, Cary Series UV-Vis Spectrophotometer), where absorbance was measured at a wavelength of 280 nm and the molar absorption coefficient was 0.667 $\mathrm{mL}\times {\mathrm{mg}}^{-1}\times {\mathrm{cm}}^{-1}$ [5]. All salt and BSA solutions were stored at 4 °C. CD spectroscopy was used to check the long-term stability of the BSA solutions over a period of 8 weeks. No changes were observed and the protein remained in its native form under these conditions. The protein buffer and salt buffer solutions studied were prepared by mixing the appropriate amounts of stock solutions with buffer just before each measurement.

### 3.2. Measuring Viscosities and Densities

The viscosity of the solutions was measured with the Ostwald microviscosimeter (Micro-Ostwald, V4 Kap I 51710 A) in a thermostatic bath. The temperature was controlled with a pre-thermostat (LAUDA DLK 10) and a thermostat (LAUDA ECO SILVERO). Measurements were made in the temperature range from 5 to 45 °C with an accuracy of ±0.01 °C. The densities were determined with oscillating U-tube densimeter (Anton Paar DMA 5000 Density meter).

### 3.3. Measuring Particle Sizes and Zeta Potentials

Particle sizes and zeta potentials were all measured using an Anton Paar particle size analyzer (Litesizer 500). Particle size and zeta potential were determined by measuring the dynamic light scattering (DLS) and electrophoretic light scattering (ELS) of the sample. DLS measurements were set to 15 1-min runs, with equilibration time set to 5 min. The particle size was evaluated from the volume distribution. Zeta potential measurements were repeated 3 times, with each of them consisting of 100 runs. The Smoluchowski approximation was used to process the results, considering the viscosity of a given salt-buffer solution. We used QS High Precision Cells with a light path of 10 × 10 mm from Hellma Analytics and a Univette from Anton Paar. All sample solutions were filtered through 0.22 μm Sartorius Ministart Syringe Filter before measurements. Due to the high concentration of our protein solutions, measurements of particle size and zeta potential should be evaluated primarily from the point of view of the influence of the chemical nature of the salt ions (ion-specific effects).

## 4. Results and Discussion

### 4.1. Viscosity of Aqueous-Buffer BSA Solutions

**Figure 1:**
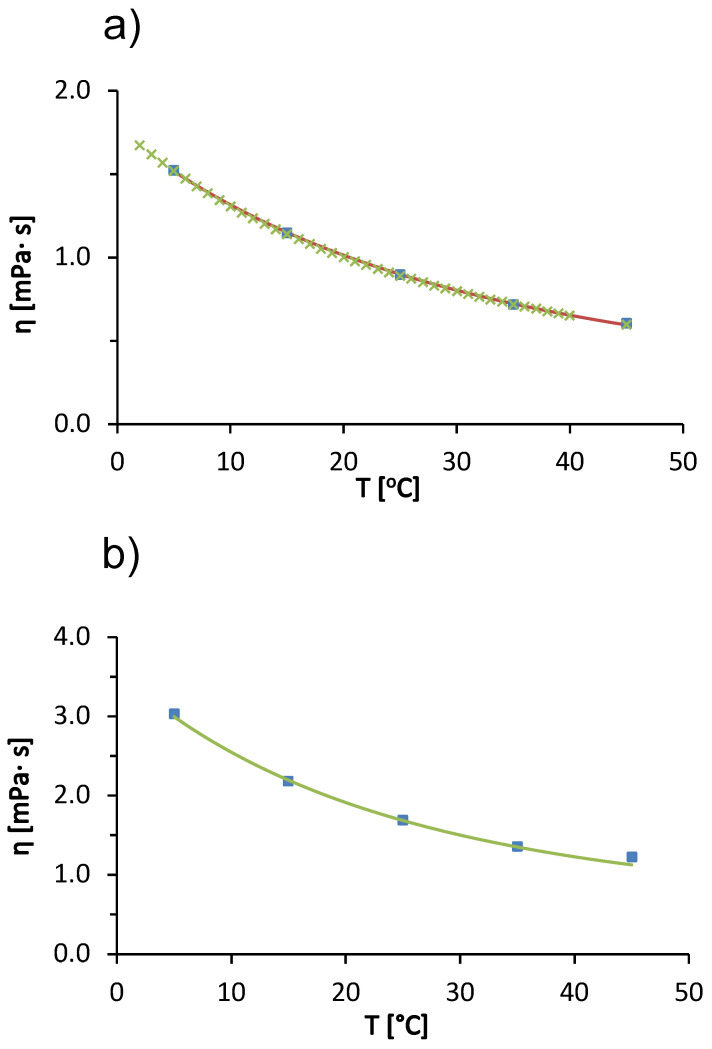

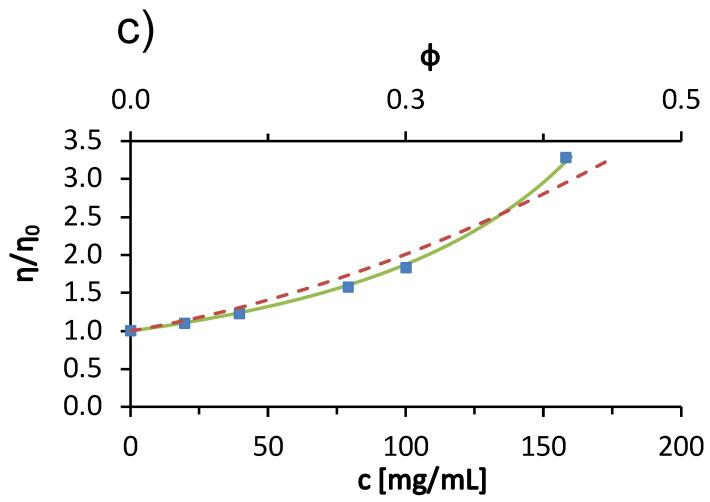
(**a**) Temperature dependence of viscosity of pure water (green symbols) and of 20 mM aqueous acetate buffer (pH = 4.3; blue symbols; absolute measurement error ±0.004 mPa×s). The solid line represents a modified Arrhenius function fit (Equation (6)). (**b**) Temperature dependence of 100 mg/mL BSA solution in acetate buffer (symbols) and the corresponding modified Arrhenius function fit (solid line; Equation (3); absolute measurement error ±0.002 mPa×s). (**c**) Concentration dependence of relative viscosity for the aqueous BSA buffer solution at 25 °C (symbols). The solid line represents a modified Arrhenius function fit and the dashed line corresponds to the hard-sphere model fit (Equation (2); the upper x-axes denote the volume fraction of protein represented as a hard sphere; the absolute measurement error was ±0.003 mPa×s). Model parameters are given in Table 1 and in the text.

We begin our discussion with the temperature trends in the viscosity of the solvent (20 mM acetate buffer with pH = 4.3) used to prepare the protein-salt solutions and for pure water. In Figure 1a we see that the viscosity of the acetate buffer decreases with increasing temperature. The viscosity of the buffer at a given temperature is almost identical to the viscosity of pure water (the differences are within experimental uncertainty). The decrease in viscosity of the liquid with increasing temperature is a consequence of the weakening of intermolecular interactions between water molecules. As the temperature increases, the thermal energy of the water molecules increases, which causes the hydrogen bonding network of the water molecules in the liquid state to gradually break down [29]. In Figure 1a, the solid line corresponds to the modified Arrhenius model function (*cf*. Equation (6)). We see that a fit describes the experimental values of the acetate buffer very well. The fitting parameters ($B_{b}$, $D_{b}$ and $\Delta E_{b}$) are given in Table 1, together with the literature values for pure water from Monkos [11]. Since the concentration of the buffer is relatively low, the parameters for the buffer solution and for pure water are very similar. The values of the parameters for the pure acetate buffer were used in the calculation of the parameters of the Arrhenius model ($\Delta E_{p}$, $B_{p}$, $D_{p}$) of the BSA solution (Equation (3)).

Figure 1b shows the temperature trends of the viscosity of a 100 mg/mL BSA solution in acetate buffer. The viscosity of the solution decreases with increasing temperature. The cause of the temperature trend is the same as for the pure acetate buffer solution; due to the higher thermal energy of the particles in the solution, the intermolecular forces between the molecules tend to weaken. The observed effect is probably caused by the solvent; however, temperature also affects protein-protein interactions (these interactions become weaker with increasing temperature) [29].

Comparing the values of the viscosity of pure buffer and the protein-buffer solution at a given temperature (Figure 1a,b, respectively), we find that the presence of the protein significantly increases the viscosity of the solution. The viscosity of the BSA solution was always higher than the viscosity of the 20 mM acetate buffer at all temperatures measured. It is common for the solute acts as an obstacle in the solution and slow down the flow of the solution. For large macromolecular solutes, the solute may also be rotating while the fluid is flowing, and some of the energy that allows the fluid to flow is transferred to the rotating solute. This slows the flow of the fluid and increases the viscosity [30].

Measurements of the viscosity of BSA solutions were made only up to a temperature of 45 °C. Above this temperature, the BSA denatured thermally and did not return to its native state [11]. The solution changed color from yellow to white-gray, transitioned to a gel state, and measurements with the capillary viscosimeter were no longer possible. The concentration trends of viscosity of BSA solutions in acetate buffer at 25 °C are shown in Figure 1c. We see that the viscosity of the solution increases as the concentration of the protein increases. The increasing amount of protein means that there are more obstacles to the flow of the solution (excluded volume effect) and more of the energy responsible for the flow of the solution is lost in the rotational degrees of freedom of the macromolecule. The other reason for increased viscosity of the solution at increased protein concentration is due to the more frequent protein-protein interactions and the potential formation of soluble aggregates at higher protein concentration. Protein aggregation is known to significantly increase solution viscosity [30].

Figure 1c shows two model functions: (1) a modified Arrhenius model (solid line, Equation (3)) and (2) a hard-sphere model (dashed line, Equation (2)). The fitting parameters of the modified Arrhenius model for BSA solution in acetate buffer ($\Delta E_{p}$, $B_{p}$, $D_{p}$) are summarized in Table 1. The literature values for BSA in pure water are also given in Table 1. Our values and those in the literature [11] are qualitatively simmilar. However, there are minor differences in the reported values mainly due to the following: (i) different values for the pH of the solution (we used acetate buffer with pH = 4.3 and Monkos used pure water). The net charge of the protein molecule depends on the pH and affects the protein-protein interactions. (ii) Different batches of BSA used. It is well known that the properties of the solution may depend on the manufacturing batch. Even different batches from the same manufacturer may differ slightly. And (iii) we used a value of 66.43 kDa for the BSA molecular weight, while Monkos used a slightly smaller value of 66 kDa.

In Figure 1c, the dashed line represents the hard sphere model for the concentration dependence of the protein solution viscosity (the upper *x*-axis indicates the volume fraction of protein molecules.). We obtained the best fit by considering the radius of the hard sphere (Stokes radius) of the protein molecule as a fitting parameter. A value of 4.099 nm was determined. This value is slightly larger than the commonly reported experimental Stokes radii, e.g., 3.4 nm determined by fluorescence anisotropy [31] and 3.9 nm [32] determined by quasielastic light scattering. Jachimska et al. reported the hydrodynamic Stokes radius of BSA determined from diffusion coefficients measured by DLS as a function of pH. For a 1 mg/mL BSA in buffer solution with added NaCl (ionic strength of 0.15 mol/L), the radius obtained from the entire scattering peak was 4.3 nm and that calculated from the maximum value (volume average) was 3.4 [33]. The differences in the values were attributed to the fact that the BSA solution contains a significant fraction of protein aggregates. Since our measurements are for larger concentrations, where the fraction of soluble aggregates is larger, the large hard-sphere radius reflects protein aggregates. However, from the dashed line in Figure 1c, we conclude that the hard-sphere model does not describe the concentration dependence of the viscosity of the BSA solution as well as the modified Arrhenius function. This is mainly due to the non-spherical shape of the BSA molecule—the protein is not a hard sphere, but a heart-shaped globular protein.

### 4.2. Dependence of the Viscosity, Particle Size and Zeta Potential of Aqueous-Buffer BSA Solutions on the Concentration of Added Low Molecular Weight Salts

**Figure 2:**
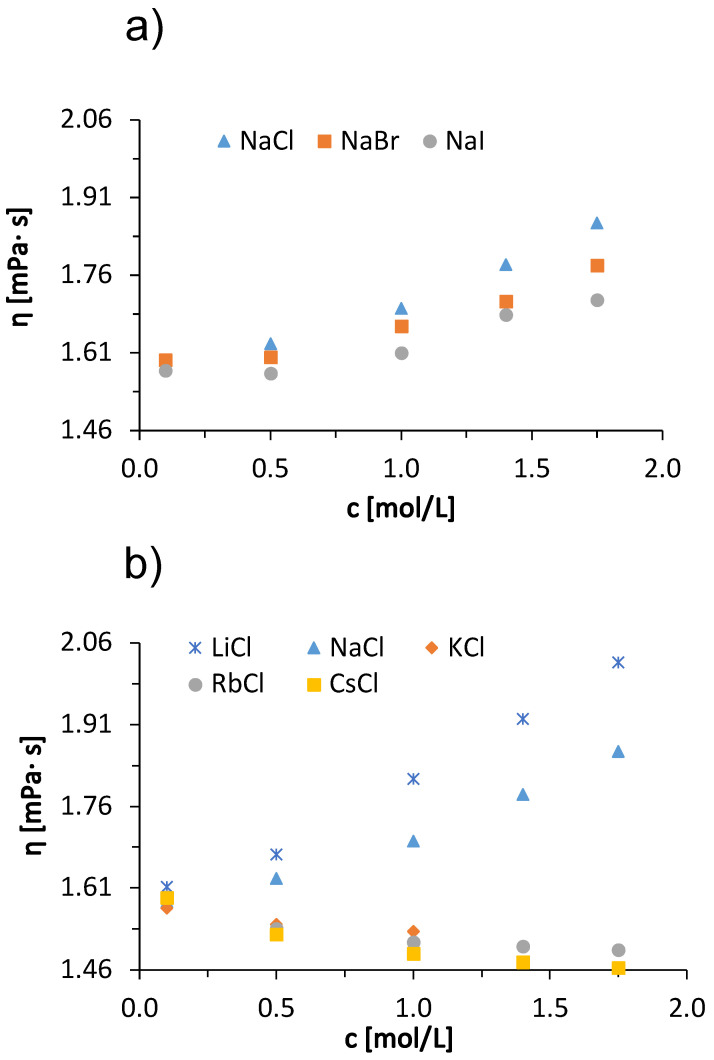
Dependence of the viscosity of 100 mg/mL aqueous-buffer BSA solution on the concentration of added (**a**) sodium halides (NaCl, NaBr, NaI) and (**b**) alkali chlorides (LiCl, NaCl, KCl, RbCl, CsCl) at 25 °C with absolute measurement error ±0.002 mPa×s.

Next, we describe the viscosity trends of BSA solutions in acetate buffer with added low molecular weight salts. Figure 2 shows the viscosity of the 100 mg/mL BSA solutions at 25 °C as a function of salt concentration. Measurements were made for salt concentrations of 0.1, 0.5, 1.0, 1.4, and 1.75 mol/L. The only exception was KCl, where measurements were made up to 1 mol/L because of the relatively low solubility of KCl. In panel (a), results are given for added sodium halides (NaCl, NaBr, NaI), whereas data in panel (b) are for alkali chloride salts (LiCl, NaCl, KCl, RbCl, CsCl). Significant salt-specific effects can be observed. The viscosity of the solution increased with increasing concentration of the added LiCl, NaCl, NaBr and NaI salts, while it decreased with the addition of KCl, RbCl and CsCl salts. Similar salt-specific trends in viscosity are also observed in protein-free salt buffer and salt water solutions [34]. Although the molar concentration of salt in the solution exceeds the molar concentration of BSA (∼1.5 $\times 10^{-3}$ mol/L) by about 10–100-fold, the mass concentrations of salt and protein are of the same order of magnitude (for example, 1 mol/L solution of LiCl is 42.4 mg/mL and of CsCl is 168.4 mg/mL; the concentration of BSA was 100 mg/mL). This confirms the fact that the changes in the viscosity of the solution are due to the presence of salt ions.

The trend of viscosities of solutions with added sodium halides of a certain concentration follows the so-called direct Hofmeister series (Cl^−^ > Br^−^ > I^−^), while the viscosities of BSA solutions with added alkali chlorides follow the so-called indirect Hofmeister series (Li^+^ > Na^+^ > K^+^ > Rb^+^ > Cs^+^). There is a large viscosity drop between the NaCl and KCl buffer BSA solutions (Figure 2b). This large difference can be explained by the sign change of the Jones-Dole viscosity *B*-coefficient (see Table 2). According to their influence on the hydration water ions are divided into kosmotropes and chaotropes. Kosmotropic ions have positive *B*-coefficient values and chaotropic ions have negative *B*-coefficient values. For solutions with NaCl, the *B*-coefficient value is 0.080 L/mol, while for KCl it is −0.014 L/mol. Na^+^ is considered a weakly kosmotropic ion, while K^+^ is a weakly chaotropic ion.

**Figure 3:**
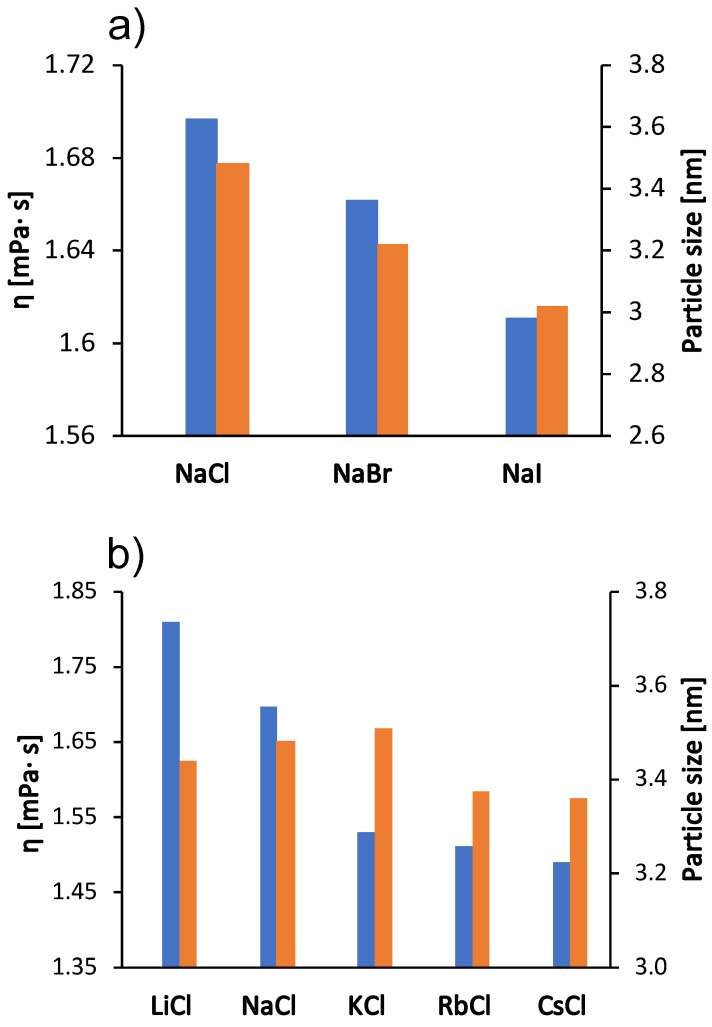
Viscosity trends at 25 °C for the 100 mg/mL aqueous-buffer BSA solutions with added 1 mol/L low molecular weight salt (blue bars, left *y*-axes) and protein size (hydrodynamic radius) in 0.5 mol/L salt solutions (red bars, right *y*-axes). (**a**) Sodium halides (NaCl, NaBr, NaI) and (**b**) alkali chlorides (LiCl, NaCl, KCl, RbCl, CsCl). The absolute error was ±0.002 mPa×s for viscosity and ±0.8 nm for particle size.

**Figure 4:**
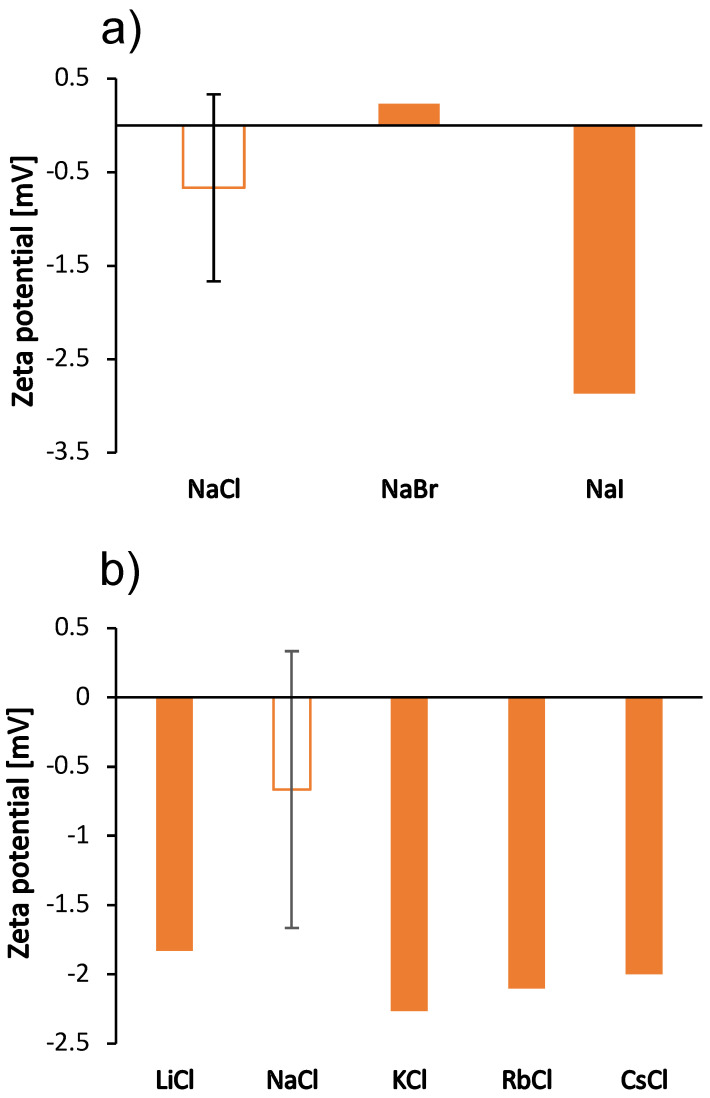
Zeta potential of BSA molecules in the 100 mg/mL aqueous-buffer solutions with added 0.5 mol/L low molecular weight salt. (**a**) Sodium halides (NaCl, NaBr, NaI) and (**b**) alkali chlorides (LiCl, NaCl, KCl, RbCl, CsCl). The value of the zeta potential of the salt-free BSA solution was 5.2 mV. Note that the ionic strength of the salt-free solution is different from the ionic strength of the solutions with added salt. Except for BSA-NaCl solution, the absolute error was estimated to be ±0.9 mV.

We were interested in how these kosmotropic/chaotropic properties of the ions affect their interaction with protein molecules. We therefore determined the dependence of the size of the BSA molecule (the hydrodynamic radius) on the type of salt (the size indicating the protein aggregation propensity) as well as the value of the zeta potential for the protein molecular surface. The results are shown in Figure 3 and Figure 4 for the case when the salt concentration was 1 mol/L and 0.5 mol/L, respectively. The results in the case of sodium salts (panel a) are in agreement with the viscosity measurements: The viscosity of the solutions is greater as the size of the protein species in the solutions increases (Stokes-Einstein equation). Since BSA molecules are positively charged at pH 4.3, cations do not readily interact with the protein surface. The changes in protein particle size for chloride salts (panel b) are small and do not follow the trend of viscosity. The viscosity of chloride salts is probably dominated by their effect on the solvent structure.

Since the salt ions are too small to affect the size of the protein by binding to its surface, we have assumed that the change in size is a consequence of the formation of protein aggregates. This has been observed previously in the determination of the cloud point temperature of hen’s egg white lysozyme solutions [34] and of BSA [23]. To further investigate the effect of salt ions on the aggregation tendency, we measured the zeta potential of the solutions in the presence of various salts. The results are shown in Figure 4; the zeta potential follows the same trend as the viscosities for sodium salts. As salt anions bind to surface-charged sites, they decrease the net charge of proteins and make them more prone to aggregation. Our results for the zeta potential of sodium salts are consistent with this prediction: The largest protein species were observed in the solutions with zeta potential values close to zero, which occur in the case of mostly kosmotropic ions. These ions are strongly hydrated and therefore less prone to binding. As mentioned earlier, cations of chloride salts do not interact well with the positive surface of BSA and the changes in zeta potential values of their solutions are minimal. BSA solutions with added NaCl did not give reliable results for zeta potential due to blackening of the electrode.

There is an interesting comparison between our experimental results on the influence of salts on the viscosity of BSA solutions and the results of L. Medda et al. [8]. These authors measured the diffusion coefficient of BSA solutions with added salts. They obtained the reverse trend for the diffusion coefficient of BSA solutions for sodium halides and a bell-shaped trend for alkali chlorides. Viscosity and diffusion coefficient (*D*) are correlated with the Stokes-Einstein equation ($D=k_{B}T/\left(6\pi r\eta \right)$, where $k_{B}$ is Boltzmann’s constant and *T* is the absolute temperature). Our trends for BSA solutions with added sodium halides correlate well with the reported trends in the diffusion coefficients of BSA. On the other hand, the addition of alkali chlorides resulted in a non-monotonic (bell-shaped) trend in the diffusion coefficient, which was not consistent with the trends in the viscosities of BSA solutions with added alkali chlorides. The difference in the results could be due to the experimental conditions: We measured viscosity at 25 °C prepared in an acetate buffer at pH = 4.3, with a BSA concentration of 100 mg/mL and 1 mol/L salt, whereas in the study by Medda et al., the conditions were 37 °C in water (pH = 7) with a BSA concentration of 40 mg/mL and with 0.1 mol/L salt added [8]. We suspect that the effect of alkali cations is more sensitive to the experimental conditions than that of halide anions. At our pH, the net charge of BSA is positive, whereas at pH = 7, the net charge is negative. The subtle influence of cations is more pronounced in solutions where the macromolecule has a net negative charge. It has been shown that anions have much more pronounced ion-specific effects than cations and that these become important as the net negative charge of the protein increases [35].

**Table 2:** Viscosity *B*-coefficients [36] and polarizabilities [37] of selected ions at 25 °C.

Ion	*B*-Coefficient	Polarizability in Water, α(0) [Å]
Li^+^	0.146	0.0285
Na^+^	0.085	0.1485
K^+^	−0.009	0.7912
Rb^+^	−0.033	1.3411
Cs^+^	−0.047	2.2643
Cl^−^	−0.005	3.764
Br^−^	−0.033	5.068
I^−^	−0.073	7.409

### 4.3. Temperature Dependence of Viscosity of Aqueous-Buffer BSA Solutions with Added Low Molecular Weight Salts

**Figure 5:**
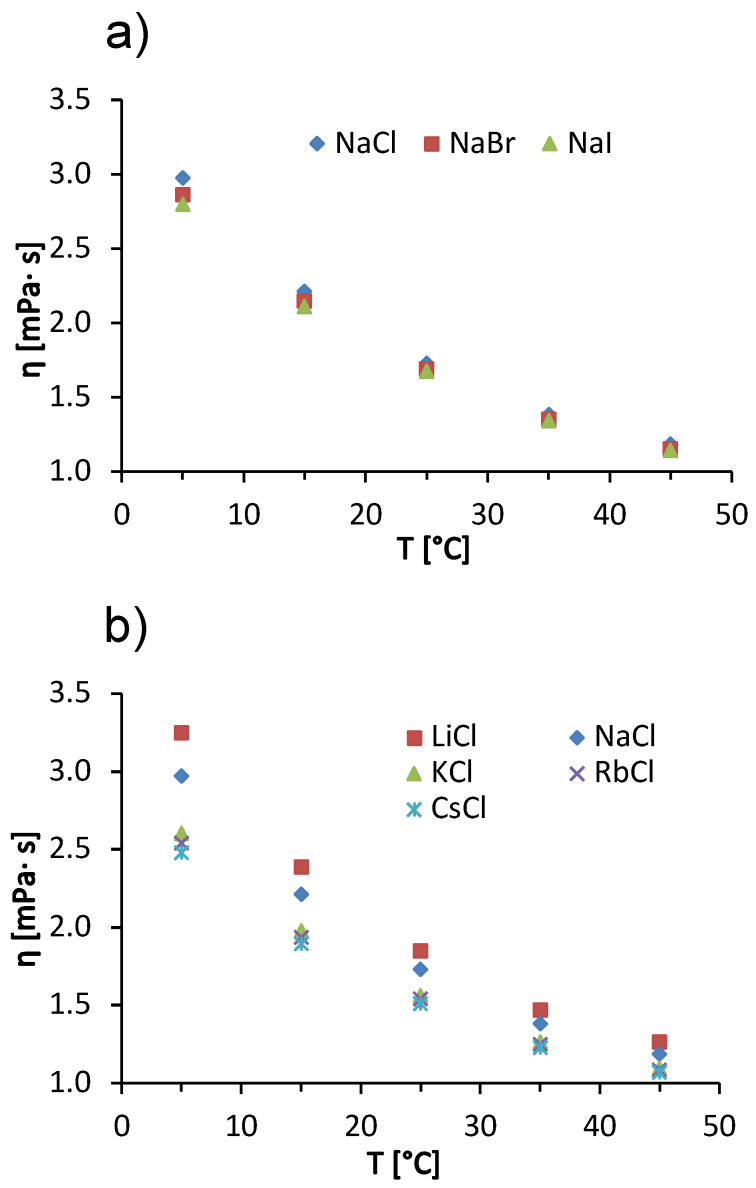
Temperature dependence of 100 mg/mL aqueous-buffer BSA solutions with added 1 mol/L low molecular weight salt. (**a**) Sodium halides (NaCl, NaBr, NaI) and (**b**) alkali chlorides (LiCl, NaCl, KCl, RbCl, CsCl) with absolute measurement error ±0.005 mPa×s.

In addition to the concentration effects of salts on solution viscosity, we also discuss the influence of temperature on ion-specific effects. In Figure 5, we show the temperature trends of the viscosity of a 100 mg/mL BSA solution in acetate buffer with added 1 mol/L salts. The data for added sodium halides are given in panel (a) and for alkali chlorides in panel (b). In the interval of measurements, the viscosity of BSA salt solutions decreases with increasing temperature. The reason for this is the same as mentioned earlier when explaining the temperature trends of pure buffer and salt-free BSA solutions (*cf*. Figure 1a,b). Both for solutions with added alkali chlorides and for sodium halides, the differences between the viscosities of BSA salt solutions decrease with increasing temperature. This effect is most clearly seen in Figure 5b, where a significant difference between the viscosities of BSA solutions with added NaCl and with added KCl can be observed at 5 °C, while the difference becomes almost invisible at 45 °C. This effect is probably a consequence of the temperature on the water structuring around the ions. At 45 °C, the thermal energy is high enough to reduce the difference in solvation of the kaotropic and cosmotropic ions—as the temperature increases, the structure of the water breaks down, regardless of the characteristic of an ion. This means that the viscosities of the solutions do not depend significantly on the identity of the added salt [38]. In our case, we have two kosmotropic ions (Li^+^ and Na^+^) and three chaotropic ions (K^+^, Rb^+^, and Cs^+^). The kosmotropic ions attract the negative dipole of the water molecules around them more strongly and form an ordered water structure around them. This allows the protein molecules to interact with each other and to form larger aggregates, increasing the viscosity of the solution [38]. The effect decreases with increasing temperature. The Hofmeister trends at other temperatures are the same as at 25 °C, we have a direct Hofmeister series for sodium halides (Figure 5a) and an indirect Hofmeister series for alkali chlorides (Figure 5b).

## 5. Conclusions

The focus of this study was on the ion-specific effects on the viscosity of BSA solutions in acetate buffer with pH = 4.3 at different temperatures and salt concentrations. A direct Hofmeister series for the anions of the sodium halide salts was determined for the solution viscosity, particle sizes, and zeta potential (Cl^−^ > Br^−^ > I^−^). An inverse Hofmeister series applied to the cation viscosities of the added alkali chlorides (Li^+^ > Na^+^ > K^+^ > Rb^+^ > Cs^+^), but not to the particle sizes and zeta potentials. Under the conditions studied, the pH of the solutions was below the isoionic point of the protein and therefore the macromolecule was net positively charged. For this reason, the anions preferentially interacted with the protein surface, resulting in shielding of the surface charge (decrease in zeta potential) and formation of protein aggregates (increase in particle size). No significant differences were measured when comparing the chloride salts. The inverse Hofmeister series for cations is probably a consequence of their different interaction with the solvent molecules but not with the protein surface. An extension of the study to pH values above the isoionic point, where BSA molecules carry a net negative charge, would be required to shed more light on the effect of cations. Ion-specific effects were more pronounced at lower temperatures and for larger salt concentrations. These trends correlate well with trends in the polarizabilities of the ions in question and with the Jones-Dole *B*-coefficients of the corresponding aqueous salt. A hard-sphere protein model was not very successful in describing the dependence of solution viscosity on protein concentration, but indicates the importance of the excluded volume effect in high protein concentrations. A much larger hard-sphere radius than the Stokes radius of the BSA had to be used to give a reasonably qualitative account of the experimental data. This could be explained by our observation that BSA molecules form aggregates in solution. On the other hand, a semi-empirical Arrhenius model function proved to be very well suited to model the concentration dependence of the viscosity of the solution. Further extension of the theory to include the salt effect would be required to account for the ion-specific effects. To apply a more contemporary statistical mechanical theory of protein solution viscosity, the dependence of the hydrodynamic radius of the molecule on the identity of the added salt would need to be determined more precisely.

## Figures and Tables

**Table 1:** Parameters of the modified Arrhenius model function for: 20 mM acetate buffer with pH = 4.3 (Equation (6)), pure water (*cf*. Ref. [11]), BSA solution in acetate buffer with pH = 4.3 (Equation (3)) and BSA solution in pure water (*cf*. Ref. [11]).

Parameter	20 mM Acetate Buffer	Water
$B_{b}$	24.378	25.94
$D_{b}$ [$K^{-1}$]	0.01751	0.02
$\Delta E_{b}$ [$\mathrm{kJ}\times {\mathrm{mol}}^{-1}$]	30.103	32.01
$B_{p}$	$3.709\times 10^{5}$	$3.891\times 10^{5}$
$D_{p}$ [$K^{-1}$]	639.64	648.8
$\Delta E_{p}$ [$\mathrm{kJ}\times {\mathrm{mol}}^{-1}$]	$4.924\times 10^{5}$	$5.374\times 10^{5}$
$\nu_{b}$ [$m^{3}\times {\mathrm{kg}}^{-1}$]	$2.043\times 10^{-3}$	$1.417\times 10^{-3}$

## Data Availability

The data presented in this study are all given in the present article.

## References

[1] Varlan A., Hillebrand M. (2010). Bovine and human serum albumin interactions with 3-carboxyphenoxathiin studied by fluorescence and circular dichroism spectroscopy. Molecules.

[2] Tian Z.Y., Song L.N., Zhao Y., Zang F.L., Zhao Z.H., Chen N.H., Xu X.J., Wang C.J. (2015). Spectroscopic study on the interaction between naphthalimide-polyamine conjugates and bovine serum albumin (BSA). Molecules.

[3] Zhang L., Cai Q.Y., Cai Z.X., Fang Y., Zheng C.S., Wang L.L., Lin S., Chen D.X., Peng J. (2016). Interactions of bovine serum albumin with anti-cancer compounds using a proteOn XPR36 array biosensor and molecular docking. Molecules.

[4] Grabowska O., Kogut M.M., Żamojć K., Samsonov S.A., Makowska J., Tesmar A., Chmur K., Wyrzykowski D., Chmurzyński L. (2021). Effect of tetraphenylborate on physicochemical properties of bovine serum albumin. Molecules.

[5] Bujacz A., Zielinski K., Sekula B. (2014). Structural studies of bovine, equine, and leporine serum albumin complexes with naproxen. Proteins.

[6] Sharma V., Jaishankar A., Wang Y.C., McKinley G.H. (2011). Rheology of globular proteins: Apparent yield stress, high shear rate viscosity and interfacial viscoelasticity of bovine serum albumin solutions. Soft Matter.

[7] Feng R., Konishi Y., Bell A.W. (1991). High accuracy molecular weight determination and variation characterization of proteins up to 80 ku by ionspray mass spectrometry. J. Am. Soc. Mass Spectrom..

[8] Medda L., Monduzzi M., Salis A. (2015). The molecular motion of bovine serum albumin under physiological conditions is ion specific. Chem. Commun..

[9] Bukackova M., Rusnok P., Marsalek R. (2018). Mathematical methods in the calculation of the zeta potential of BSA. J. Solut. Chem..

[10] Salis A., Boström M., Medda L., Cugia F., Barse B., Parsons D.F., Ninham B.W., Monduzzi M. (2011). Measurements and theoretical interpretation of points of zero charge/potential of BSA protein. Langmuir.

[11] Monkos K. (1996). Viscosity of bovine serum albumin aqueous solutions as a function of temperature and concentration. Int. J. Biol. Macromol..

[12] Hong T., Iwashita K., Shiraki K. (2018). Viscosity control of protein solution by small solutes: A review. Curr. Protein Pept. Sci..

[13] Tanford C., Buzzell J.G. (1956). The viscosity of aqueous solutions of bovine serum albumin between pH 4.3 and 10.5. J. Phys. Chem..

[14] Yadav S., Shire S., Kalonia D. (2011). Viscosity analysis of high concentration bovine serum albumin aqueous solutions. Pharm. Res..

[15] Harding S.E. (1998). Dilute Solution Viscometry of Food Biopolymers.

[16] Singh M., Chand H., Gupta K.C. (2005). The studies of density, apparent molar volume, and Viscosity of bovine serum albumin, egg albumin, and lysozyme in aqueous and RbI, CsI, and DTAB aqueous solutions at 303.15 K. Chem. Biodivers..

[17] Kunz W., Henle J., Ninham B.W. (2004). ‘Zur Lehre von der Wirkung der Salze’ (about the science of fthe effect of salts): Franz Hofmeister’s historical papers. Curr. Opin. Colloid Interface Sci..

[18] Kunz W. (2009). An attempt of a general overview. Specific Ion Effects.

[19] Kunz W. (2010). Specific ion effects in colloidal and biological systems. Curr. Opin. Colloid Interface Sci..

[20] Lo Nostro P., Ninham B.W. (2012). Hofmeister phenomena: An update on ion specificity in biology. Chem. Rev..

[21] Okur H.I., Hladilkova J., Rembert K.B., Cho Y., Heyda J., Dzubiella J., Cremer P.S., Jungwirth P. (2017). Beyond the Hofmeister series: Ion-specific effects on proteins and their biological functions. J. Phys. Chem. B.

[22] Madeira P.P., Rocha I.L., Rosa M.E., Freire M.G., Coutinho J.A. (2021). On the aggregation of bovine serum albumin. J. Mol. Liq..

[23] Džudžević Čančar H., Belak Vivod M., Vlachy V., Lukšič M. (2022). Phase stability of aqueous mixtures of bovine serum albumin with low molecular mass salts in presence of polyethylene glycol. J. Mol. Liq..

[24] Brini E., Fennell C.J., Fernandez-Serra M., Hribar-Lee B., Lukšič M., Dill K.A. (2017). How water’s properties are encoded in its molecular structure and energies. Chem. Rev..

[25] Yang Z. (2009). Hofmeister effects: An explanation for the impact of ionic liquids on biocatalysis. J. Biotechnol..

[26] Jelesarov I., Dürr E., Thomas R.M., Bosshard H.R. (1998). Salt effects on hydrophobic interaction and charge screening in the folding of a negatively charged peptide to a coiled coil (leucine zipper). Biochemistry.

[27] Masuelli M.A., Gassmann J. (2017). Intrinsic Viscosity Bovine Serum Albumin in Aqueous Solutions: Temperature Influence on Mark-Houwink Parameters.

[28] Mendoza C.I., Santamaría-Holek I. (2009). The rheology of hard sphere suspensions at arbitrary volume fractions: An improved differential viscosity model. J. Chem. Phys..

[29] Landau L.D., Akhiezer A.I., Lifshitz E.M. (1967). General Physics: Mechanics and Molecular Physics.

[30] Saluja A., Kalonia D.S. (2008). Nature and consequences of protein–protein interactions in high protein concentration solutions. Int. J. Pharm..

[31] González Flecha F.L., Levi V. (2003). Determination of the molecular size of BSA by fluorescence anisotropy. Biochem. Mol. Biol. Educ..

[32] Park J.M., Muhoberac B.B., Dubin P.L., Xia J. (1992). Effects of protein charge heterogeneity in protein-polyelectrolyte complexation. Macromolecules.

[33] Jachimska B., Wasilewska M., Adamczyk Z. (2008). Characterization of globular protein solutions by dynamic light scattering, electrophoretic mobility, and viscosity measurements. Langmuir.

[34] Brudar S., Hribar-Lee B. (2021). Effect of buffer on protein stability in aqueous solutions: A simple protein aggregation model. J. Phys. Chem. B.

[35] Janc T., Vlachy V., Lukšič M. (2018). Calorimetric studies of interactions between low molecular weight salts and bovine serum albumin in water at pH values below and above the isoionic point. J. Mol. Liq..

[36] Jenkins H.D.B., Marcus Y. (1995). Viscosity B-coefficients of ions in solution. Chem. Rev..

[37] Tavares F.W., Bratko D., Blanch H.W., Prausnitz J.M. (2004). Ion-specific effects in the colloid-colloid or protein-protein potential of mean force: Role of salt-macroion van der Waals interactions. J. Phys. Chem. B.

[38] Goldsack D.E., Franchetto R. (1977). The viscosity of concentrated electrolyte solutions. I. Concentration dependence at fixed temperature. Can. J. Chem..

